# Rapid and sensitive isolation of *Campylobacter jejuni* using immunomagnetic separation from patient specimens exposed to oxygen

**DOI:** 10.1128/spectrum.01907-24

**Published:** 2025-02-18

**Authors:** So Yeon Kim, Young-Sun Yun, Kwang-Jun Lee, Jonghyun Kim

**Affiliations:** 1 Division of Zoonotic and Vector-borne Disease Research, Center for Infectious Diseases Research, Korea National Institute of Health, Cheongju, South Korea; 2 Division of Acute Viral Disease, Center for Emerging Virus Research, Korea National Institute of Health, Cheongju, South Korea; University of Chicago, Chicago, Illinois, USA

**Keywords:** *Campylobacter jejuni*, monoclonal antibody, magnetic bead–antibody complex, probable case, confirmed case, stool specimen, detection sensitivity

## Abstract

This study describes a method for detecting *Campylobacter jejuni* in patient stools with subsequent isolation using antibody–magnetic beads in conjunction with selective culture and PCR. Monoclonal antibodies specific for the flagellin A and major outer membrane protein of *C. jejuni* were generated; two clones (1C7 and 4B2) were used to coat magnetic beads for immunomagnetic separation (IMS). *C. jejuni* strain NCTC11168 was recovered from human stool samples spiked with varying concentrations (10^1^–10^5^ CFU/mL) by *Campylobacter* (Campy)-IMS or a conventional culture-based method and plated on modified charcoal–cefoperazone–deoxycholate agar; the number of colonies was enumerated. The detection limits of Campy-IMS and conventional culture-based method with spiked stool samples were 10^2^ and 10^4^ CFU/mL, respectively. The sensitivity of IMS-PCR was 10–10,000-fold higher than that of direct PCR. The recovery rate of *C. jejuni* from spiked stools stored for 12 to 72 h decreased from 72.3 to 5.9% with Campy-IMS and from 48.5 to 0.1% with the conventional culture-based method. Importantly, of 20 PCR (+)/bacterial culture (−) samples that were diagnosed as probable cases according to general criteria, 95% (19/20) were confirmed positive by Campy-IMS. Thus, this study suggests a solution to overcome the problems caused by the inconsistency between probable and confirmed cases of *Campylobacter* infection.

**IMPORTANCE:**

The isolation, cultivation, and maintenance of *Campylobacter* spp. are difficult because of the microaerophilic conditions and specific medium needed. Although selective media are useful for the initial isolation of *Campylobacter*, subsequent exposure of the sample to oxygen has a detrimental effect on the positive culture rate of *Campylobacter*, significantly lowering the isolation rate from patient samples. In this study, the detection limit was improved by combining immunomagnetic separation and PCR methods to quickly detect *Campylobacter jejuni* in clinical patient stool samples using antibody*–*magnetic beads. Therefore, this study is expected to improve confirmation of *C. jejuni* infection where diagnosis would previously fail with patient samples because of oxygen exposure, inappropriate diagnostic methods, and interference from other bacteria in the sample.

## INTRODUCTION

*Campylobacter* spp. are often the most common causative agent of gastroenteritis in developed countries, with an estimated 166 million people infected worldwide (1). *Campylobacter* infection has multiple potential sources (2–4); in particular, a high risk of infection occurs with handling raw poultry and eating undercooked chicken. *Campylobacter* are the second leading cause of known food poisoning due to bacterial pathogens in Korea, with approximately 800 cases reported in 2015 (5, 6). *Campylobacter* spp. identification from patient stool samples is difficult because of the specific media and culture conditions required. The isolation and cultivation of *Campylobacter* spp. are difficult because they require a microaerophilic environment and a specific medium; moreover, maintaining this pathogen in culture is more difficult compared to other gram-negative bacteria (7). The most problematic aspect is that the positive culture rate of *Campylobacter* spp. decreases in inverse proportion to the time the sample is exposed to oxygen (8).

Although guidelines have been established for isolating *Campylobacter* from human stool specimens (9), the method used by public health laboratories can vary since the conventional culture-based method is not always effective despite being considered the gold standard. Selective medium is useful for initially isolating *Campylobacter* but can yield uninterpretable results due to problems, such as exposure of specimens to the atmosphere and low initial bacterial counts. Additionally, under oxygen and temperature stress, *Campylobacter* spp. may enter a viable but nonculturable (VBNC) state in which traditional culture techniques fail (10). Therefore, a more rapid and reliable technique for detecting and identifying *Campylobacter* spp. is desired.

Molecular biological and antigen/antibody (Ab)-based methods have been used to improve assay sensitivity, which has not been confirmed for conventional diagnostic methods (11). Methods used by public health laboratories to detect pathogenic bacteria have shifted toward PCR, which is usually more rapid, sensitive, and specific compared to conventional culture-based methods (12). However, in these approaches, determining the effects of various factors present in the specimens can be difficult. Moreover, a PCR-based diagnosis alone is inadequate for determining the optimal treatment course for patients, collecting epidemiological data, or identifying correlations with the source of contamination. Therefore, *Campylobacter* spp. must still be isolated via bacterial culture.

Immunomagnetic separation (IMS) has been applied to bacterial detection (13). Target bacteria bound to the functionalized magnetic bead (MB) surface are isolated by inoculating the beads in a medium selective for the target organism. IMS is also used for rapid diagnosis in combination with PCR-based detection (14–16). This method is advantageous as this removes potential inhibitory factors from the reaction and increases the accuracy of diagnosis by concentrating the target organism in the sample (13). Thus, Ab–MB complexes (AMBc) only capture target bacteria, while others are removed in the washing step. The detection and isolation limits can be improved by selectively concentrating the target bacteria and removing factors that can inhibit the PCR reaction during the IMS process.

In this study, we developed a rapid and sensitive isolation and detection method by combining *Campylobacter* (Campy)-IMS with PCR for the identification of live *Campylobacter jejuni* in human stool specimens.

## MATERIALS AND METHODS

### Bacterial strains and culture conditions

The strains used in this study are listed in Table 1. *C. jejuni* subsp. *jejuni* strain NCTC 11168 was used as a standard reference for *C. jejuni* isolation. The NCTC11168 inoculum was grown under microaerobic conditions (5% O_2_, 10% CO_2_, and 85% N_2_) at 42°C for 48 h in Mueller–Hinton broth (MHB). The absorbance of the inoculum at 600 nm was measured with a GeneQuant 1300 spectrophotometer (GE Healthcare, Uppsala, Sweden) and adjusted to 1.0 to obtain a bacterial cell concentration of 4.5 × 10^8^ CFU/mL. This concentration was confirmed by plating bacteria on mCCDA or blood agar. Other bacterial species (*Salmonella* Typhimurium, *Vibrio pharaemolyticus*, *Shigella sonnei*, and *Escherichia coli*) were grown on tryptic soy broth. A bacterial suspension in MHB with a concentration of 2 × 10^9^ CFU/mL was prepared per strain.

**TABLE 1:** Characteristics of bacterial strains in this study

Bacterial strain	Source	Year	Characteristics
NCTC11168	ATCC^*a*^		O:2
Wild-type *C. jejuni* 13-2237	Stool	2017	Isolated from patients
Wild-type *C. jejuni* 13-2452	Stool	2017	Isolated from patients
Wild-type *C. jejuni* 13-2628	Stool	2017	Isolated from patients
Wild-type *C. jejuni* 13-2706	Stool	2017	Isolated from patients
Wild-type *C. jejuni* 14-97	Stool	2017	Isolated from patients
Wild-type *C. jejuni* 14-515	Stool	2017	Isolated from patients
Wild-type *C. jejuni* 14-582	Stool	2017	Isolated from patients
Wild-type *C. jejuni* 14-929	Stool	2017	Isolated from patients
Wild-type *C. jejuni* 14-953	Stool	2017	Isolated from patients
Wild-type *C. jejuni* 14-954	Stool	2017	Isolated from patients
*E. coli* DH5α	Invitrogen		F-Ф80/Z△M15△(*lac*ZYA-*arg*F) U169 *rec*A1 *end*A1 *hsd*R17 (rK−, mK+) *pho*A supE44 λ-thi-1 *gyr*A96 *rel*A1
*Salmonella* Typhimurium	Stool	2011	Group 0:9(D1)-[1],9,12H,g,m/-
*Vibrio pharahaemolyticus*	Stool	2008	O3:K29
*Shigella sonnei*	Stool	2010	*inv, ipa*
Shiga toxin-producing *E. coli* (STEC)	Stool	2010	*stx*1, *stx*2, O157:H7
Enterotoxigenic *E. coli* (ETEC)	Stool	2004	ST, LT, O6
Enteropathogenic *E. coli* (EPEC)	Stool	2008	*eae*A

^*a*^ ATCC, American Type Culture Collection.

### Gene cloning and expression of *C. jejuni* recombinant proteins

The *porA* gene encoding major outer membrane protein (MOMP) and the *flaA* gene encoding flagellin A (FlaA) were amplified from purified chromosomal DNA of *C. jejuni* NCTC11168 with primers Momp-F (5ʹ-GGCCGGATCCAATGAAACTAGTTAAACTTAGTTTA-3ʹ) and Momp-R (5ʹ-GGCCAAGCTTTTAGAATTTGTAAAGAGCTTGAAG-3ʹ), flaA-F (5ʹ-GGCCGGATCCGGATTTCGTATTAACACAAATGTTGCAGCA-3ʹ), and flaA-R (5ʹ-GGCCAAGCTTTTGTAATAATCTTAAAACATTTTGCTGACT-3ʹ). Amplicons were cloned into pET28 (Novagen), and insets were completely sequenced on both strands for verification. Recombinant plasmids were transformed into *E. coli* BL21(DE3). Expression of polyhistidine-tagged recombinant proteins was induced by adding 1 mM isopropyl thiogalactopyranoside to mid-exponential-phase cultures grown at 37°C. Induced *E. coli* was harvested after 3 h and lysed via sonication, and cell debris was cleared via centrifugation. Recombinant proteins were purified from cleared lysates using Ni-NTA agarose (Qiagen) (Fig. S1).

### Production of monoclonal antibodies

BALB/c mice were immunized with 100 µg of *C. jejuni* recombinant FlaA and MOMP (rFla and rMOMP, respectively) and 0.2 mL of Freund’s incomplete adjuvant (Sigma, USA). Intraperitoneal injections were administered on days 0, 14, and 28. For the final immunization, 100 µg proteins were administered through the tail vein 3 days before cell fusion. Splenocytes from immunized mice and myeloma cells (SP2/0) were collected and fused as described by De St. Groth and Scheidigger (6). Hybridomas were selected for in hypoxanthine–aminopterin–thymidine medium, and those showing significant growth after 21 days were tested for the presence of specific antibodies against rFlaA and rMOMP via an enzyme-linked immunoassay. After final confirmation, the hybridoma cell line was propagated in tissue culture and stored in liquid N_2_. Monoclonal antibodies (MAbs) were purified from ascites using saturated ammonium sulfate and stored at −20℃. The MAb subclass was determined using a mouse monoclonal antibody isotyping kit (Sigma).

### MAb preparation and MB coupling

MAbs 1C7 and 4B2 that had a high specificity and affinity against rFla and rMOMP were prepared in this study. MAbs were prepared by coating Dynabeads M-270 epoxy (Invitrogen Dynal AS, Oslo, Norway) with the mouse anti-FlaA (1C7) and anti-MOMP MAbs (clones 4B2 and 7D8) according to the manufacturer’s instructions. Briefly, 5 mg (~3.3 × 10^8^) of Dynabeads M-270 epoxy was washed with 1 mL of phosphate-buffered saline (PBS, pH 7.4), separated with a magnet, and resuspended in 100 µL of PBS. The Ab (100 µg) was added to the bead suspension, followed by incubation for 18 h at 37°C on a HulaMixer (Thermo Fisher Scientific, Waltham, MA, USA). Coated beads were washed four times with 1 mL volumes of PBS containing 0.1% bovine serum albumin (BSA) for 5 min at 4°C, then stored in 150 µL of PBS with 0.1% BSA (~2 × 10^9^ beads/mL) at 4°C.

### Stool sample collection

**Fig 1:**
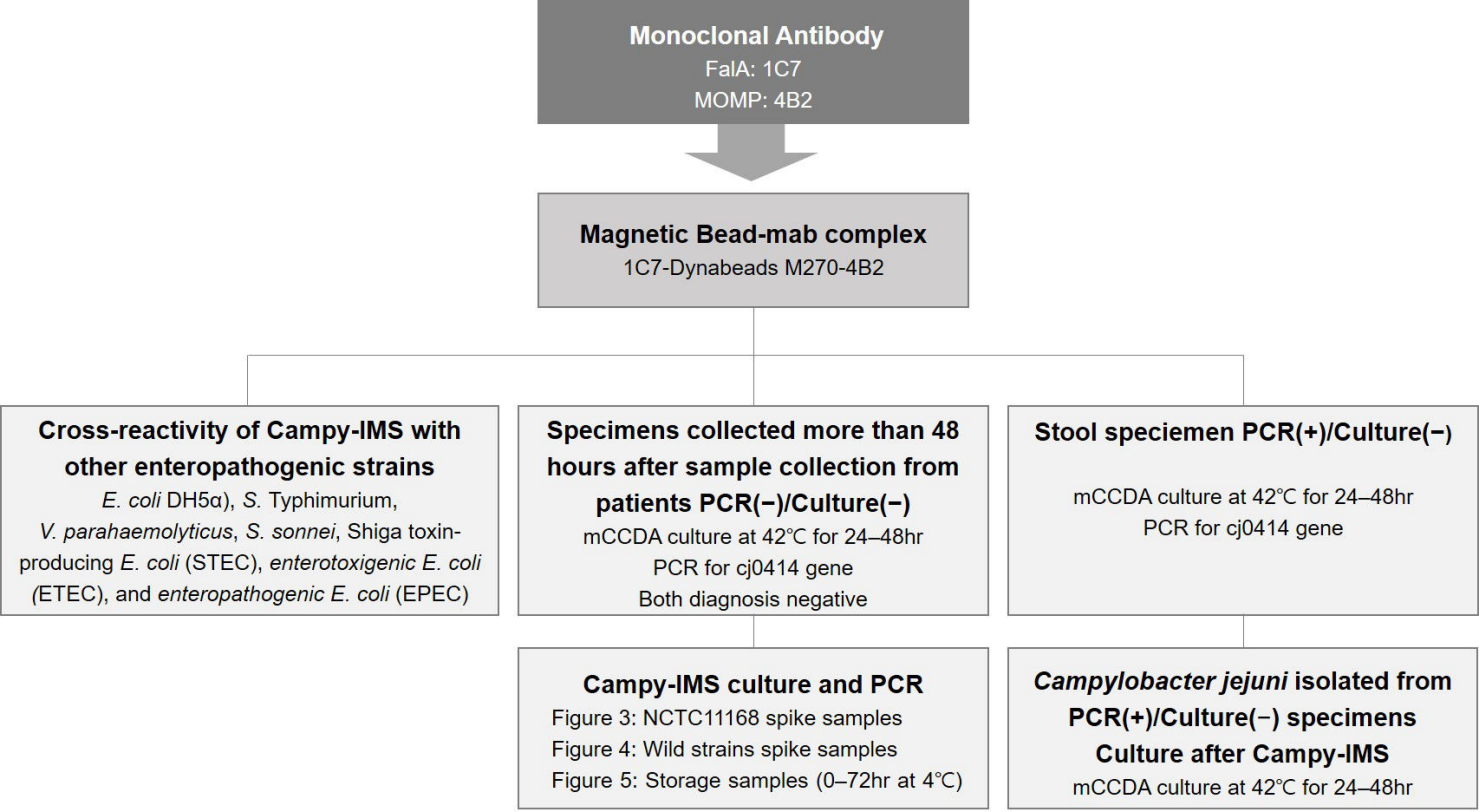
Schematic of the study workflow.

All stool samples used in this study were collected at a single stool testing institution (Chungchungbuk-do Province Institute of Health and Environment Research). Two types of stool samples were analyzed (Fig. 1). The first type of stool samples was used to confirm recovery from spiked specimens, and these all tested negative for *C. jejuni* via bacterial culture and PCR. Samples were stored at 4°C until use. The second type of stool samples was used to isolate bacteria from PCR (+)/bacterial culture (−) specimens by IMS; these samples were categorized as 2, 3, 4, or 5 days according to the time elapsed after collection. Five PCR (+)/bacterial culture (−) specimens were collected per group. Specimens were immediately used for IMS without preservation. All stool samples were obtained from patients with diarrhea identified through the EnterNet surveillance program (https://is.cdc.go.kr). All specimens were transported to the laboratory where they were immediately classified as *Campylobacter* spp. based on culture and PCR tests.

### PCR amplification

A 24 bp forward primer (5ʹ-CAAATAAAGTTAGAGGTAGAATGT-3ʹ) and 22 bp reverse primer (5ʹ-CCATAAGCACTAGCTAGCTGAT-3ʹ) targeting the *cj0414* gene of *C. jejuni* were used for PCR, yielding a 161 bp product (11). The PCR reaction volume of 20 µL contained 1 µL of each primer (20 pmole), 10 µL of ExTaq (Takara Bio, Otsu, Japan), 6 µL of double-distilled water (DDW), and 2 µL of template. The amplification was performed on a PCR Dice Gradient thermal cycler (Takara Bio). Cycling conditions were 95°C for 5 min, 30 cycles of 95°C for 30 s, 58°C for 30 s, 72°C for 30 s, and 72°C for 7 min. Products were analyzed via gel electrophoresis on a 2% (w/v) agarose gel containing SYBR Safe DNA Gel Stain (Thermo Fisher Scientific) in 0.5× TBE buffer (445 mM Tris, 445 mM boric acid, 10 mM EDTA, pH 8.0) and visualized via ultraviolet transillumination. In our experiments, all PCR tests followed the abovementioned method.

### MAb selection

Serial 10-fold dilutions of NCTC 11168 were added to 1 mL of MHB to obtain final concentrations of 10^1^–10^5^ CFU/mL. A 50 µL volume of MBs combined with the three MAbs (clones 1C7, 7D8, and 4B2) was added to MHB spiked with *C. jejuni* and incubated at room temperature for 10 min with gentle agitation. Unbound *C. jejuni* cells were removed by washing five times with PBS containing 0.01% Tween 20 (PBST). The *C. jejuni*-AMBc was resuspended in 100 µL of PBS and plated on modified charcoal–cefoperazone–deoxycholate agar (mCCDA; Oxoid, Basingstoke, UK), followed by incubation under microaerobic conditions at 42°C for 48 h. Beads coated with 1C7 and 4B2 showed the highest recovery and were used in subsequent experiments.

### Recovery of *C. jejuni* from contaminated samples

Contaminated samples were prepared by mixing 100 µL of NCTC11168 (10^1^–10^5^ CFU) and 0.9 mL of each of the other bacterial species (10^9^ CFU). To prepare spiked samples, 100 µL of each of five NCTC 11168 suspensions was added to the five stool samples (0.9 mL each). Samples were prepared in three batches: one was used for IMS, another for bacterial culture, and the third for direct PCR. A 1 mL volume of each NCTC11168-contaminated sample was incubated with Campy-IMS as described above. After IMS, beads were resuspended in 100 µL of PBS and then serially diluted and plated on selective medium. A 2 µL aliquot of the remaining beads was used as the template for IMS-PCR. Colonies arising from another spiked sample were counted according to a Korea National Research Institute of Health protocol (https://www.nih.go.kr/). Briefly, 1 mL of the spiked sample was serially diluted in mCCDA after 18 h of enrichment and cultured at 42°C for 48 h under microaerobic conditions. The other spiked sample was centrifuged at 12,000×*g* for 5 min, and the pellet was resuspended in 100 µL of DDW and boiled for 5 min to obtain crude genomic DNA, of which 2 µL was used as a template for direct PCR. The sensitivity of the reaction was defined as the lowest concentration of *C. jejuni* that yielded a positive result.

### *C. jejuni* isolation from stored stool samples

A 1.5 mL volume of NCTC11168 (1.5 × 10^6^ CFU) was mixed with 13.5 mL of stool sample. Spiked specimens were divided into 1 mL (10^5^ CFU/ml) aliquots that were stored at 4°C and sampled after 0, 12, 24, 48, and 72 h. Campy-IMS and isolation by bacterial culture were performed using samples collected after various storage times. One set of aliquots was used for direct PCR.

### *C. jejuni* isolation from PCR-positive specimens by Campy-IMS

To confirm the isolation of *C. jejuni* in PCR (+)/bacterial culture (−) samples by Campy-IMS, 0.1 g of stool sample was resuspended in 1 mL of MHB for Campy-IMS. A 1 mL volume of each MHB-diluted stool sample was combined with 50 µL of immunomagnetic beads, followed by incubation at 42°C for 6 h under microaerobic conditions with rotation. The *C. jejuni*-conjugated MBs were washed five times with PBST, resuspended in 100 µL of MHB, plated on mCCDA, and incubated at 42°C for 48 h under microaerobic conditions.

### Statistical analysis

Data were analyzed using Prism 8.3.0 software (GraphPad, Inc., San Diego, CA, USA). Differences between groups were evaluated by one-way analysis of variance. *P* < 0.05 was considered significant.

## RESULTS

### Efficiency of MAbs

**Fig 2:**
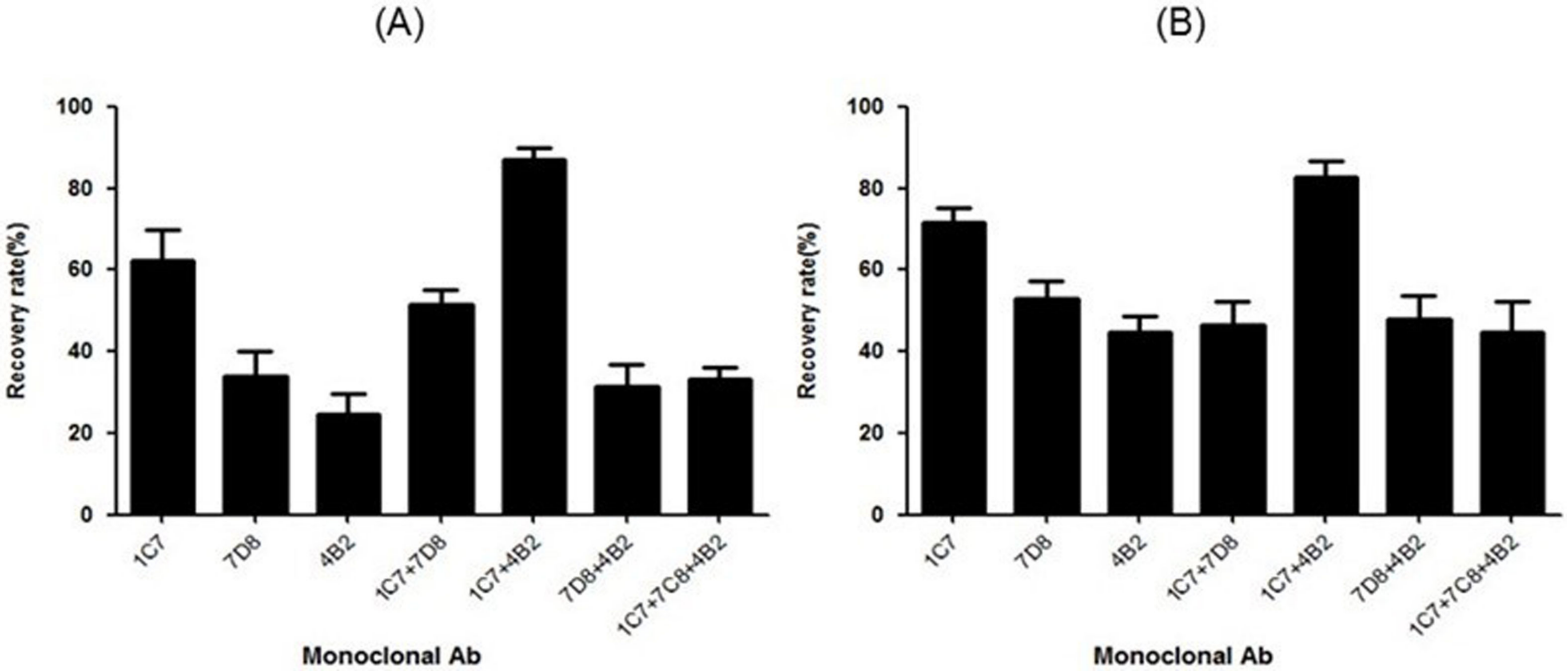
Performance of different Abs for the capture of NCTC11168. Clones 1C7, 7D8, 4B2, 1C7 + 7D8, 1C7 + 4B2, 7D8 + 4B2, and 1C7 + 7CB + 4B2 were conjugated to MBs. Recovery from samples spiked with high (A, 10^5^ CFU) and low (B, 10^3^ CFU) concentrations of NCTC11168 was evaluated. NCTC11168 and MAb-MBs were incubated at room temperature for 10 min, and unbound cells were removed by washing. NCTC11168 bound to MBs was resuspended in PBS, and serial dilutions were plated on mCCDA, followed by incubation at 42°C for 48 h. Average measurements with standard deviation from three independent experiments are shown.

Various factors determine the efficiency of IMS, of which, the most important is the quality of the capture Ab. Herein, we used MAbs against *C. jejuni* proteins FlaA and MOMP. To confirm the ability of these MAbs to capture *C. jejuni*, the isolation rate of strain NCTC11168 was evaluated using beads coated with each of the prepared MAbs. The AMBc was added to a pure bacterial sample, and the capture efficiency was quantitatively analyzed by colony counting. AMBc-harboring MAb clones 1C7, 7D8, and 4B2 had recovery rates of 62, 37, and 26%, respectively (Fig. 2). Clone 1C7 had average recovery rates of 71.5 and 62.4% at 10^5^ and 10^3^ CFU, respectively, while for clone 4B2, these were 44.7 and 24.7% at 10^5^ and 10^3^ CFU, respectively. MBs coated with two or more Abs showed that 1C7 and 4B2 were the most effective, with a recovery of >80%. Furthermore, the capture efficiency of MBs coated with a mixture of 1C7, 4B2, and 7D8 was markedly reduced, suggesting that clone 7D8 competitively inhibited clones 1C7 and 4B2. The capture efficiencies of MBs coated with a mixture of 1C7 and 4B2 were 87 and 82.7% at low and high concentrations of NCT11168, respectively (Fig. 2).

### Recovery of NCTC11168 in mixed samples by Campy-IMS

**Fig 3:**
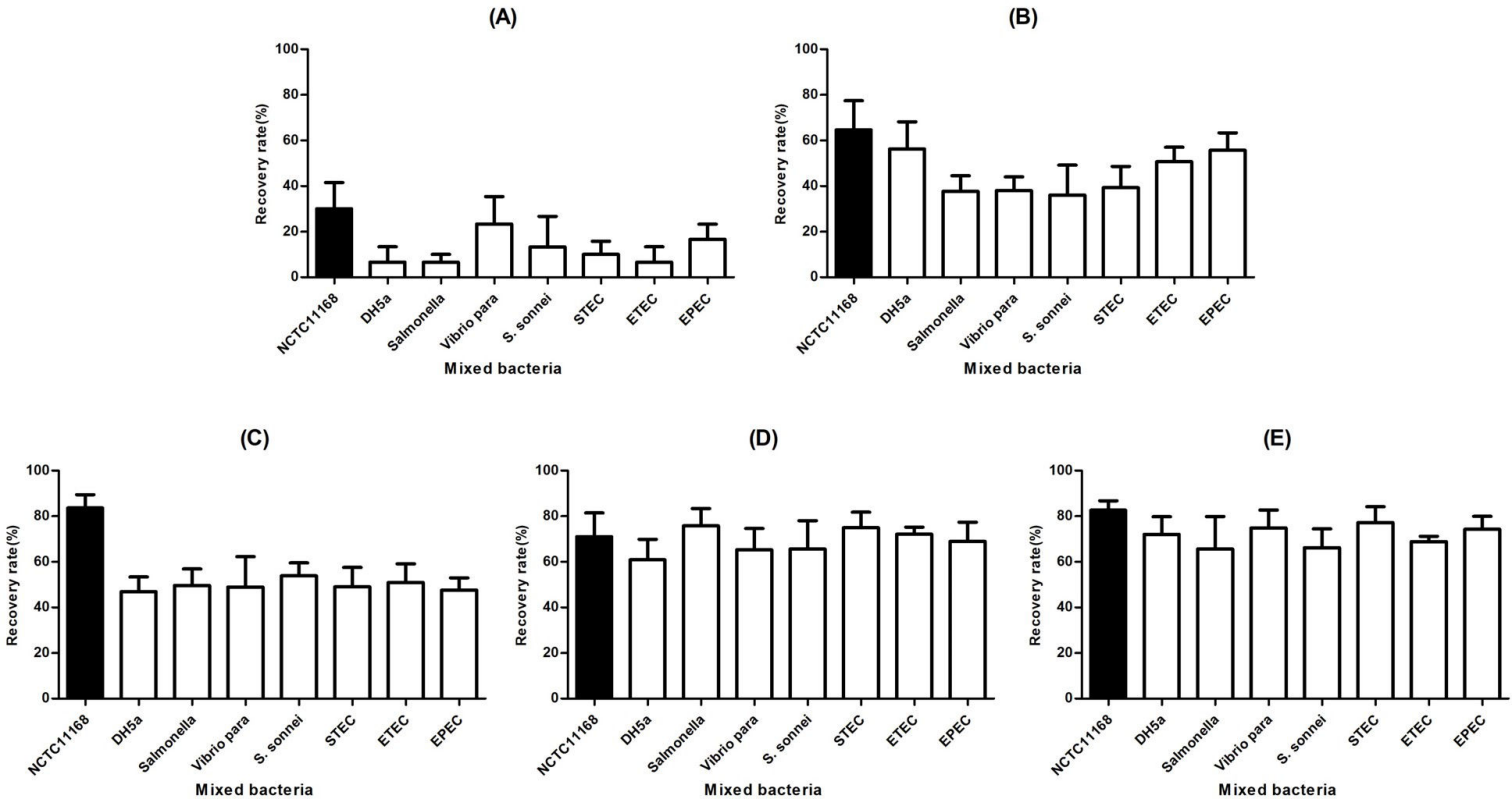
Evaluation of Campy-IMS specificity for NCTC11168 in samples containing contaminating pathogenic bacteria [*E. coli* DH5α, *S.* Typhimurium, *V. parahaemolyticus*, *S. sonnei*, STEC, enterotoxigenic *E. coli* (ETEC), and enteropathogenic *E. coli* (EPEC)]. NCTC11168 at 10^1^–10^5^ CFU was included as a control. (A–E) NCTC11168 was mixed with other bacteria at the following ratios: 10^1^:10^9^ CFU (**A**), 10^2^:10^9^ CFU (**B**), 10^3^:10^9^ CFU (**C**), 10^4^:10^9^ CFU (**D**), and 10^5^:10^9^ CFU (**E**).

To assess the cross-reactivity of the AMBc with other pathogenic bacteria, the NCTC11168 detection efficiency in samples also containing *E. coli* (DH5α), *Salmonella enterica* serovar Typhimurium, *Shigella sonnei*, *Vibrio parahaemolyticus*, Shiga toxin-producing *E. coli* (STEC), enterotoxigenic *E. coli*, and enteropathogenic *E. coli* was evaluated. The isolation rate increased from 15 to 70% as the bacterial concentration increased (Fig. 3). However, a similar isolation rate was observed for samples spiked with 10^4^–10^5^ CFU/mL *C*. *jejuni* (Fig. 3D and E).

The specificity of the AMBc was confirmed by culturing IMS-obtained beads on a blood agar plate under aerobic conditions. *E. coli* DH5α and *S*. Typhimurium were detected at rates of 2 and 1.3%, respectively (data not shown); we suspected that this was due to errors from the washing step rather than nonspecific binding. As we increased the number of washes from five to 10 times, the rate of detection of these bacteria decreased to <0.6%. Similarly, the average nonspecific binding rate of *E. coli* was between 0.36 and 0.05% at low and high spiking levels, respectively, when *Salmonella* was isolated by IMS (17). Another study used rabbit and goat anti-*Bacillus anthracis* MAbs to obtain a recovery rate for nontarget organisms of 5 and 2%, respectively, for *Bacillus cereus* spores and 4% and 20%, respectively, for *E. coli* (18). These results suggested that our Campy-IMS method has reasonable specificity in detecting low levels of NCTC11168.

### Comparison of Campy-IMS and bacterial culture

**Fig 4:**
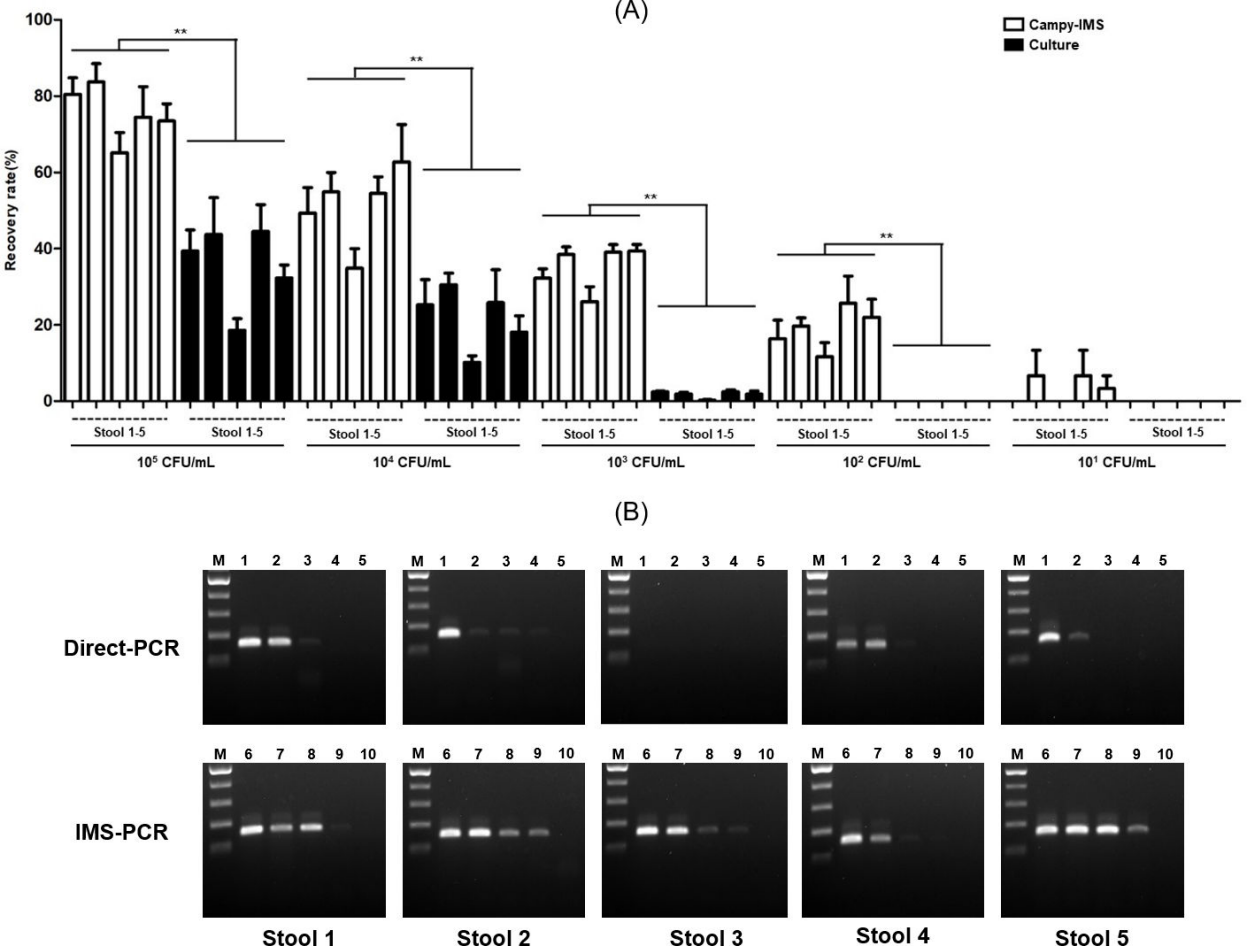
Comparison of recovery rate and detection sensitivity between IMS-PCR and conventional methods. (**A**) Comparison of recovery rates between Campy-IMS and bacterial culture from five stool samples spiked with NCTC11168 from 10^1^ to 10^5^ CFU. Campy-IMS was performed without pre-enrichment, while bacterial culture was performed according to the Korea Centers for Disease Control and Prevention Manual after 18 h of preincubation. Black, Campy-IMS; gray, bacteria culture. **P < 0.01 (unpaired t test). (**B**) Comparison of the sensitivity of direct and Campy-PCR. Lanes M: 100 bp ladder (Thermo Fisher Scientific); lanes 1–5, direct PCR at 10^5^ to 10^1^ CFU/mL; and lanes 6–10, pelleted beads of Campy-IMS from 10^5^ to 10^1^ CFU/mL.

The capture efficiency of Campy-IMS in this study was tested using five *C. jejuni* culture- and PCR-negative human stool samples spiked with NCTC11168 at concentrations ranging from 10^1^ to 10^5^ CFU/mL. The recovery rate was influenced by the initial concentration of *C. jejuni* and the stool type. At *C. jejuni* concentrations of 10^4^ and 10^5^ CFU/mL, 51.3 and 75.4% of cells, respectively, were captured by Campy-IMS (Fig. 4A). Only a small proportion of the initial concentration (35.7 and 22%) was recovered by bacterial culture. At a low initial concentration (10^1^–10^3^ CFU/mL), the recovery rate showed minimal change via Campy-IMS (Fig. 4A) but decreased to 1.8% of the initial concentration of 10^3^ CFU/mL via bacterial culture (as compared with 35% by Campy-IMS), with zero recovery from several samples. The low isolation efficiency of NCTC11168 from stool sample 3 suggested that this specimen contained certain factors that inhibited bacterial binding by Abs than the others. Thus, the Campy-IMS method developed in this study may have a low detection limit for *C. jejuni* in human specimens. The complex composition of stools may be associated with higher concentrations of factors that inhibit the antigen–Ab reaction and PCR than in other types of specimens (19).

The removal of these inhibitors was then determined using IMS-PCR. Following IMS, samples were resuspended in 100 µL of PBS, and a 2 µL volume was used as a template for PCR. As a control, 1 mL of spiked feces was centrifuged, and the pellet was suspended in 100 µL of DDW, with 2 µL used for PCR. The sensitivity of IMS-PCR was 10- to 10,000-fold higher than that of direct PCR (Fig. 4B). Stool sample 3 was negative by direct PCR in samples spiked with 10^5^ CU/mL, whereas the detection limit was increased by >1,000 fold by IMS-PCR. These results demonstrate that the latter is an efficient method for rapid screening of *C. jejuni* and eliminates interference effects in the stools that inhibit target capture by the MAb, as well as in the PCR reaction.

**Fig 5:**
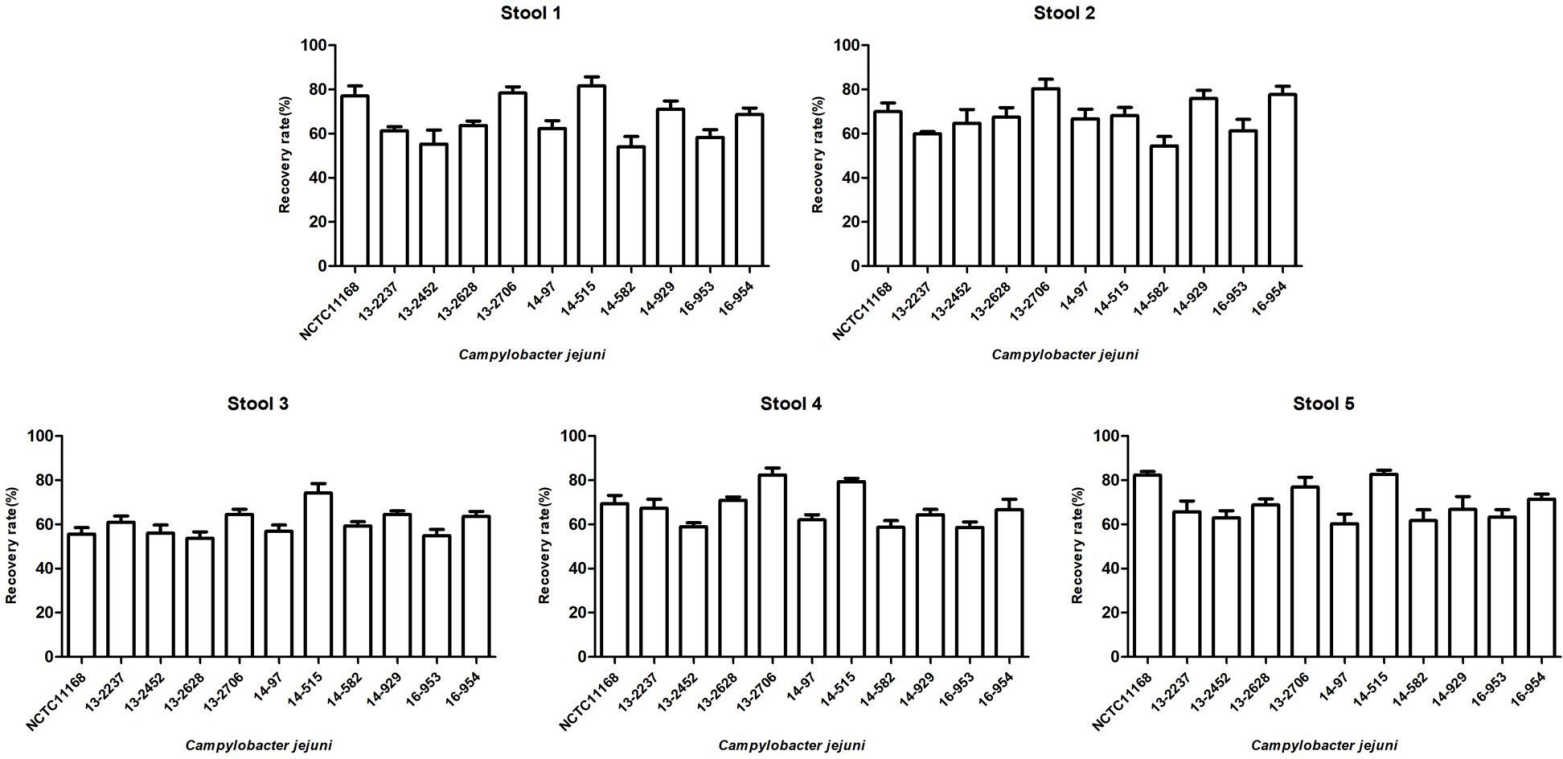
Detection of wild-type *C. jejuni* in spiked stool samples. The recovery of the target strain by Campy-IMS from stool samples spiked with 10^5^ CFU/mL wild-type *C. jejuni* isolated from patients between 2012 and 2016 was evaluated.

To determine whether Campy-IMS can capture wild-type *C. jejuni* strains other than the reference strain, 10 isolates collected through the EnterNet program from May 2017 to September 2017 were tested. The recovery rate of nonreference strains was between 53.7 and 74.2%, which was similar to that of NCTC11168 (Fig. 5).

### Recovery from stored stool samples

**Fig 6:**
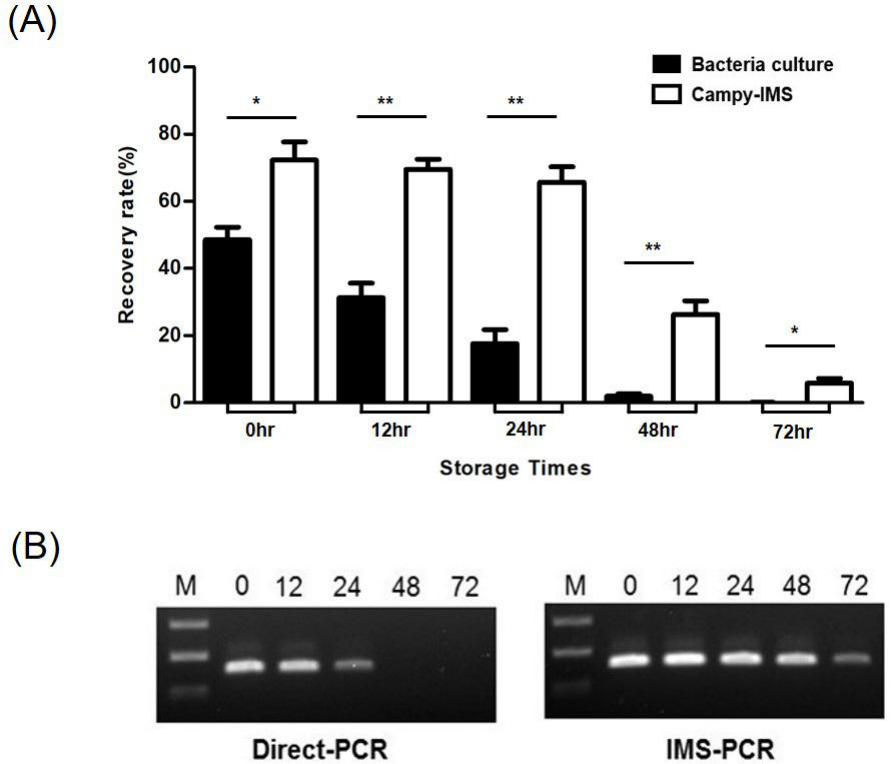
Detection of NCTC11168 in spiked stool samples stored at 4°C. (**A**) Comparison of Campy-IMS and bacterial culture. Spiked specimens were prepared by mixing NCTC11168 (10^5^ CFU) with stool samples. (**A**) Stored samples were collected at 0, 12, 24, 48, and 72 h and isolated by the two methods (black, bacterial culture; white, Campy-IMS). *P < 0.05, **P < 0.01 (unpaired *t*-test). (**B**) Sensitivity of direct PCR vs. IMS-PCR. Samples for PCR were collected in the same manner as in panel (**A**). Lane 1, 100 bp ladder (Thermo Fisher Scientific); lane 2, sample stored for 0 h; lane 3, sample stored for 12 h; lane 4, sample stored for 24 h; lane 5, sample stored for 48 h; and lane 6, sample stored for 72 h.

*C. jejuni* can enter a VBNC state in response to various environmental stressors (20–22). Several studies have reported that *C. jejuni* can survive in fecal samples from broiler chicken flocks for 90–120 h (23). We compared the recovery of NCTC11168 via bacterial culture and Campy-IMS from spiked specimens stored under aerobic conditions at 4°C for 72 h. The recovery rate from samples stored for 12–72 h decreased from 72.3 to 5.9% via Campy-IMS and from 48.5 to 0.1% via bacterial culture (Fig. 6A). The rates for samples stored up to 24, 48, and 72 h were 2.7-, 13.4-, and 91.8-fold lower via bacterial culture than via Campy-IMS.

NCTC11168 was not detected by direct PCR in samples stored for >24 h, whereas positive results were obtained by IMS-PCR for 72 h samples (Fig. 6B). The sensitivity of IMS-PCR was 100-fold higher than that of direct PCR. Diagnostic sensitivity is critical because the number of bacteria excreted through stools varies according to the time of onset of infection and patient symptoms. Our results demonstrate that Campy-IMS and IMS-PCR are more sensitive than bacterial culture for detecting NCTC11168 in stool samples containing varying ratios of culturable/nonculturable cells.

### Detection efficiency in PCR-positive and bacterial culture-negative human stool samples

**Fig 7:**
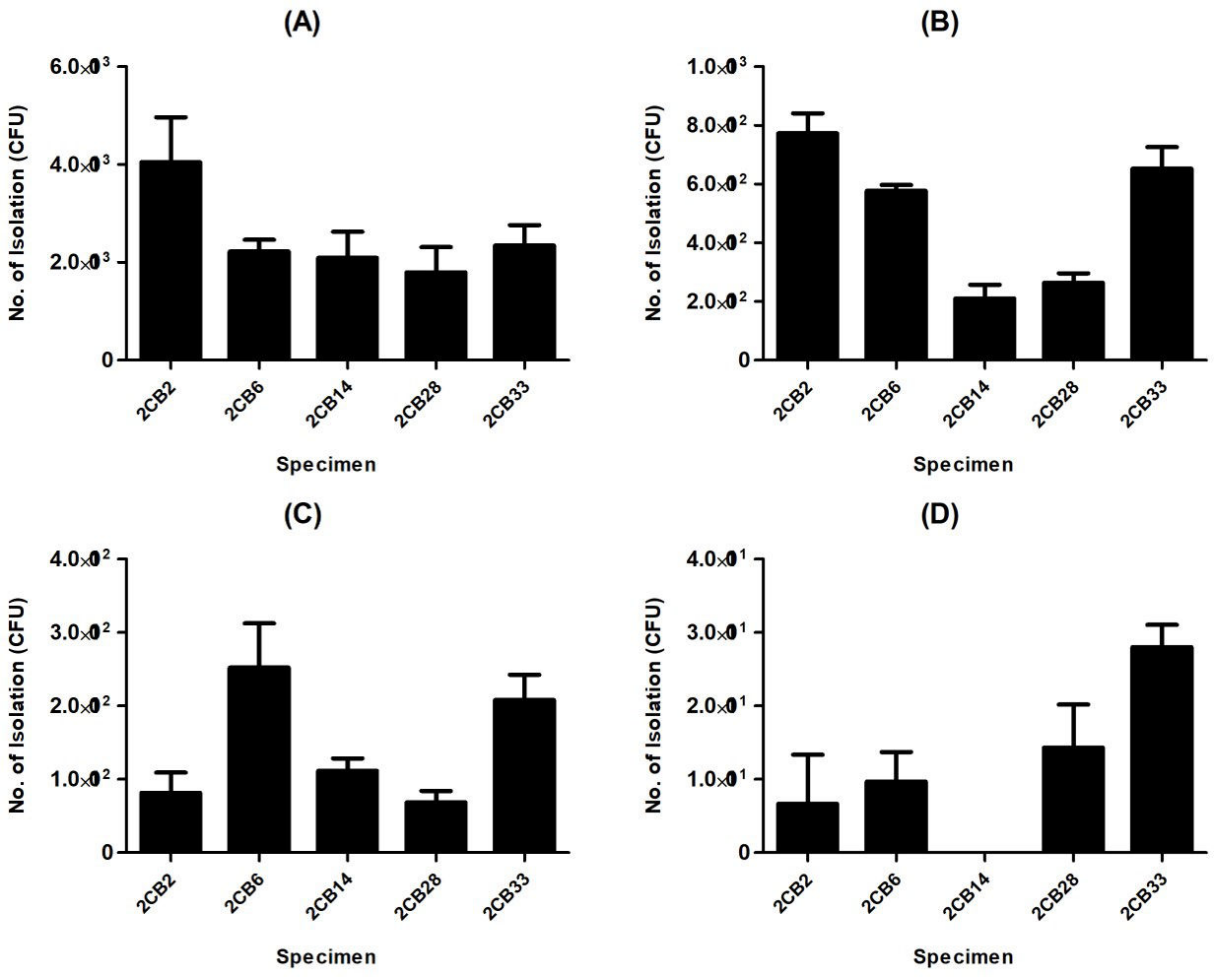
Isolation of *Campylobacter jejuni* in PCR (+)/bacterial culture (−) human samples by Campy-IMS. Stool specimens were analyzed using conventional culture and PCR. Specimens in which the *Campylobacter* spp. gene was detected but yielded no cultured bacteria were classified into groups of 48–120 h from the time of specimen collection from the patient. Among these specimens, five were used in the experiment. Samples were designated as 48 (**A**), 72 (**B**), 96 (**C**), and 120 (**D**) h according to the time elapsed after collection.

In many cases, the rates of positivity by bacterial culture and PCR are inconsistent (24, 25). To confirm the efficacy of Campy-IMS for isolating *C. jejuni* in PCR (+)/bacterial culture (−) samples, analysis was performed within 48–120 h of collection from patients with gastrointestinal illness. Interestingly, *C. jejuni* was isolated from all but one sample (5CB32, a sample exposed for 120 h); however, as the time of exposure to the atmosphere and low temperature increased, the number of isolated bacteria decreased markedly (Fig. 7). Approximately 1.8–4 × 10^3^ and 6.9 × 10^1^ to 2.5 × 10^2^ CFU/mL were isolated from samples exposed to conditions of stress for 48 and 96 h, respectively. Surprisingly, 7–28 CFUs were detected via Campy-IMS in four out of five samples exposed to oxygen for 5 days. This suggests that Campy-IMS can overcome the discrepancy between PCR and bacterial culture results.

## DISCUSSION

Bacterial cultures have been widely used to detect pathogenic bacteria but are time-consuming and labor-intensive and have low sensitivity. Recently, rapid and highly sensitive automated gene detections, such as PCR, real-time PCR, quantum dots, and aptamer detection, have been developed as alternatives (14, 15, 26, 27). This has created a new problem regarding discrepancies between bacterial culture and gene detection results. The criterion for diagnosing gastroenteritis is the isolation of pathogenic bacteria either as a confirmed case (e.g., detection of *C. jejuni* or *E. coli* in a clinical specimen) or as a probable case (i.e., one with an epidemiologic link that is confirmed by other laboratory methods, such as PCR) (17). The main factors contributing to the difference in results obtained via the two methods are target bacteria concentrations below the detection/isolation limit and the presence of inhibitors (e.g., normal flora, lipopolysaccharide, polysaccharide, proteinase K, and dead cells) in the sample. A high concentration of live target bacteria increases the isolation rate by bacterial culture and reduces the false positive rate in PCRs. Removal of complex inhibitors in the sample can increase the detection limit for both bacterial culture and PCRs, and IMS is the most suitable tool to date for this purpose.

Many studies have investigated the possibility of shortening the preconcentration process that is performed to attain the minimum number of cells required for accurate diagnosis (28, 29). In this study, we established a method for rapidly isolating and detecting *C. jejuni* while avoiding interference from multiple factors. We isolated two MAbs (1C7 and 4B2) against *C. jejuni* and covalently conjugated these with MBs. The isolation limits for *C. jejuni* in spiked stool samples were 10^2^ and 10^4^ CFU/mL for the IMS method and culture, respectively. Yu et al. (30) demonstrated a detection limit of *C. jejuni* in ground poultry meat using polyclonal Ab-coated MBs of 10^3^ CFU/g with enrichment and 10^4^ CFU/g without this. Olsvik et al. (31) demonstrated a detection limit of *C. jejuni* in chicken meat by IMS of at least 5 × 10^3^ CFU/g, which is similar to the values obtained for other pathogenic bacteria using this method (5, 6, 32).

In stools spiked with *C. jejuni* at concentrations > 10^4^ CFU/sample, the difference in recovery rate by the two methods was approximately 2-fold but increased to 19.4-fold at low concentrations (10^3^ CFU/sample). This implies that Campy-IMS is a more efficient isolation method for samples contaminated with low levels of *C. jejuni*. Infected humans excrete approximately 10^6^–10^9^ CFU/g in feces (5, 6), although a lower number of bacteria may be excreted at the early stage of infection or during the recovery period. Campy-IMS is more effective than bacterial culture in the acute stage since the amount of samples collected from patients can be limited.

Previous studies have used a pre-enrichment step (33, 34) to overcome the low numbers of contaminating bacteria, which is longer for *C. jejuni* than for other pathogenic bacteria because of the slow growth. However, without enrichment, only approximately 30 min was required for capturing the antigen by Campy-IMS and washing away other unbound bacteria. These results suggest that Campy-IMS is more efficient than bacterial culture for the rapid and sensitive detection of *C. jejuni* in specimens with low levels of contamination.

PCR is only possible if the bacterial cells are concentrated in a volume suitable for the reaction. Inhibitors in contaminated samples can preclude the detection of target bacteria by PCR. Previously, the main solutions to such interference were pre-enrichment and DNA purification. As an alternative, IMS is a simple and powerful method for the capture, recovery, and concentration of target microorganisms in samples and for the removal of other substances (normal flora, polysaccharides, and lipids) that interfere with target bacterial growth and PCR-based detection (35). In this study, IMS and PCR were combined to minimize the effects of inhibitors present in the samples. The sensitivity of IMS-PCR and removal of inhibitors were increased from 10- to 10,000-fold compared with direct PCR by increasing the initial concentration of bacteria (Fig. 4B); moreover, the conversion of *C. jejuni* to a VBNC state tended to increase during standard detection procedures due to exposure to oxygen and low-temperature (10). The IMS process concentrated *C. jejuni* cells to exceed the detection limit for PCR (Fig. 6B). The results of recent studies using IMS in combination with molecular methods for detecting foodborne pathogens indicate that the detection limit is from 10^1^ to 10^3^ CFU/mL or CFU/g (13, 36, 37), which is consistent with our finding that 10^2^ CFU/mL of *C. jejuni* can be detected in spiked stool samples without enrichment or DNA purification. Another advantage of our method is that the use of MB-bacteria complexes as PCR templates without cell lysis does not affect PCR reactions (38).

Bacteria and viruses are the most common causes of foodborne illness (39). However, stool cultures are only positive in <40% of cases (38, 40). Considerable efforts have been made to reduce the number of undiagnosed cases, which has promoted the development of highly sensitive PCRs. However, this has created new problems due to the difference in sensitivity between genomic detection methods and bacterial cultures. Clinical laboratories are gradually moving away from bacterial cultures in favor of PCRs, which are commonly used for the detection of *Campylobacter* spp. and STEC but have increasingly been used for *Shigella* and *Salmonella* in recent years (15). This trend has been attributed to the characteristics of each bacterial species. Bacterial culture is thought to be inefficient for *Campylobacter* spp. as these enter a nonculturable state in response to external stress. In 20 positive stool specimens stored at 4°C, *C. jejuni* was recovered in just 50% of samples on the day after the first sampling; and in two of samples, the bacteria survived for 12–20 days (41). In the current study, we sought to reduce the gap between confirmed and probable cases by concentrating surviving cells to a value above the detection limit by using Campy-IMS. Using spiked and cultured negative stools, we demonstrated that *C. jejuni* could be efficiently concentrated and isolated by Campy-IMS. All PCR (+)/bacterial culture (−) samples were probable cases according to the general criteria, but our method confirmed that 95% (19/20) of these were positive (Fig. 7). The detection of *C. jejuni* will improve as PCR methodologies improve (41, 42). However, PCR results do not always reflect live cells, that is, the risk of false positives increases with PCR sensitivity, which can cause unnecessary treatment of the patient and impact policy decisions that are made based on erroneous public health data.

### Conclusions

Our study presents a solution to the incongruence present between probable and confirmed cases of campylobacteriosis. The method presented herein is more efficient than culture-based methods and more sensitive than PCR while still retaining specificity. Furthermore, Campy-IMS detected *C. jejuni* from both fresh and stored stool specimens. The findings of this study are expected to help reduce the number of confirmed cases where *C. jejuni* infections fail to be diagnosed from patient specimens because of the effects of oxygen exposure and inappropriate diagnostic methods and the interference caused by the presence of PCR inhibitors. Additional studies are needed to evaluate the performance of Campy-IMS with regard to food, environmental, and livestock samples.

## SUPPLEMENTAL MATERIAL

Fig. S1 spectrum.01907-24-s0001.tif SDS-PAGE analysis FlaA and MOMP proteins.

ASM does not own the copyrights to Supplemental Material that may be linked to, or accessed through, an article. The authors have granted ASM a non-exclusive, world-wide license to publish the Supplemental Material files. Please contact the corresponding author directly for reuse.
