# Supplementary figures and images for: Rapid and sensitive isolation of Campylobacter jejuni using immunomagnetic separation from patient specimens exposed to oxygen
Source: Microbiol Spectr. 2025 Feb 18;13(4):e01907-24. doi: 10.1128/spectrum.01907-24 (PMC11960043; doi:10.1128/spectrum.01907-24)

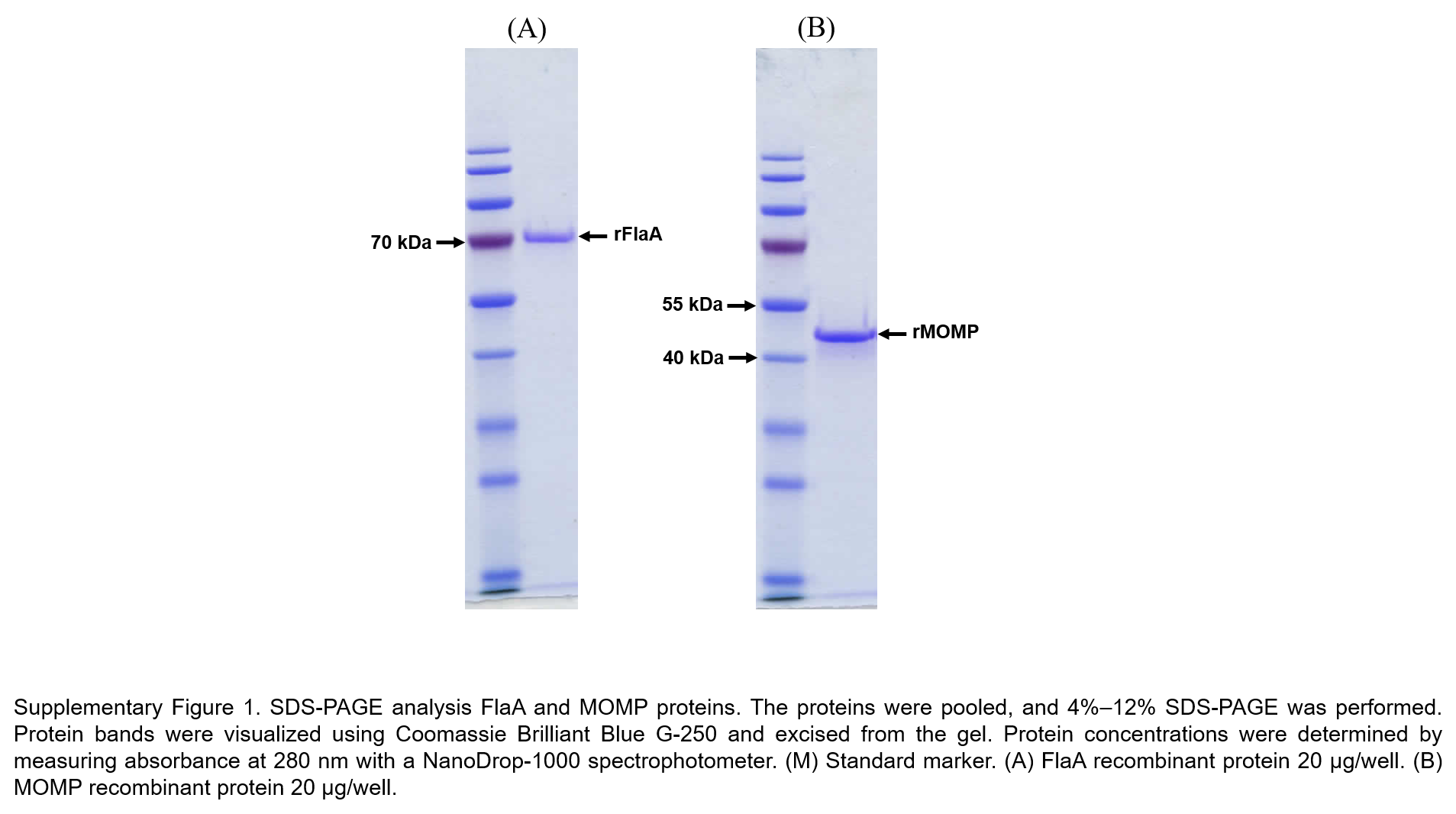

Supplement: Fig. S1 — SDS-PAGE analysis FlaA and MOMP proteins. [file spectrum.01907-24-s0001.tif]
